# Real‐life effectiveness of first‐line anticancer treatments in stage IIIB/IV NSCLC patients: Data from the Czech TULUNG Registry

**DOI:** 10.1111/1759-7714.13679

**Published:** 2020-10-05

**Authors:** Kristian Brat, Monika Bratova, Jana Skrickova, Magda Barinova, Karolina Hurdalkova, Milos Pesek, Libor Havel, Leona Koubkova, Michal Hrnciarik, Jana Krejci, Ondrej Fischer, Milada Zemanova, Helena Coupkova, Martin Svaton

**Affiliations:** ^1^ Department of Respiratory Diseases University Hospital Brno Brno Czech Republic; ^2^ Faculty of Medicine Masaryk University Brno Czech Republic; ^3^ Institute of Biostatistics and Analyses, Ltd. Brno Czech Republic; ^4^ Department of Pneumology University Hospital Pilsen Pilsen Czech Republic; ^5^ Faculty of Medicine Charles University in Prague Pilsen Czech Republic; ^6^ Department of Respiratory Medicine Thomayer Hospital Prague Czech Republic; ^7^ Department of Pneumology University Hospital Motol Prague Czech Republic; ^8^ 2nd Faculty of Medicine Charles University Prague Czech Republic; ^9^ Department of Pneumology University Hospital Hradec Kralove Hradec Kralove Czech Republic; ^10^ Faculty of Medicine Charles University in Prague Hradec Kralove Czech Republic; ^11^ Department of Pneumology and Thoracic Surgery Bulovka Hospital Prague Czech Republic; ^12^ Department of Respiratory Medicine University Hospital Olomouc Olomouc Czech Republic; ^13^ Faculty of Medicine Palacky University Olomouc Czech Republic; ^14^ Department of Oncology General Teaching Hospital Prague Czech Republic; ^15^ 1st Faculty of Medicine Charles University Prague Czech Republic; ^16^ Clinic of Comprehensive Cancer Care Masaryk Memorial Cancer Institute Brno Czech Republic

**Keywords:** Anticancer treatment, non‐small cell lung cancer, progression‐free survival, real‐life effectiveness, tyrosinkinase inhibitors

## Abstract

**Background:**

Data regarding real‐life effectiveness of any treatment may improve clinical decision‐making.

The aim of this study was to evaluate real‐life effectiveness of tyrosin‐kinase inhibitors, bevacizumab and pemetrexed as first‐line treatments in patients with advanced/metastatic non‐small cell lung cancer (NSCLC).

**Methods:**

We analyzed data of 2157 patients of the Czech TULUNG Registry of patients with advanced/metastatic NSCLC who received modern‐era treatments between 2011 and 2018. Patients treated with gefitinib, erlotinib, afatinib, bevacizumab (+ maintenance), pemetrexed (+ maintenance) as first‐line therapy were included in the study. A systematic literature search separately identified clinical trials suitable for calculation of comparator pooled OS and PFS for each regimen. For each subgroup, basic characteristics and survival data (Kaplan‐Meier estimates) are shown. We propose the “index of real‐life effectiveness” (IRE), a ratio of real‐life OS/PFS and comparator pooled OS/PFS. Univariate and multivariate logistic regression identified factors were associated with longer OS (ie, IRE>1.1).

**Results:**

Survival analysis showed median OS of 23 months for erlotinib, 29.3 months for afatinib, 19.6 months for gefitinib, 12.2 months for pemetrexed, 17.5 months for pemetrexed maintenance, 15.8 months for bevacizumab and 15.8 months for bevacizumab maintenance. Calculated IREs for OS for the regimens were: erlotinib 1.013, afatinib 1.184, gefitinib 0.736, pemetrexed 1.188, pemetrexed maintenance 1.294, bevacizumab 1.178, and bevacizumab maintenance 1.189. Multivariate regression analysis showed that these factors were associated with longer OS: lower PS for afatinib; lower PS, absence of adverse events and female sex for bevacizumab; and lower PS and female sex for pemetrexed.

**Conclusions:**

This study clearly demonstrated that real‐life effectiveness of certain treatment regimens may strongly differ in various populations/health care systems, and comparison between TULUNG data and pooled survival data from trials showed higher real‐life effectiveness for most of the studied first‐line regimens. Lower ECOG PS, younger age, female sex and adverse events were associated with longer survival in most regimens.

**Key points:**

**Significant findings of the study:**

Comparison between TULUNG data and pooled survival data from trials showed higher real‐life effectiveness for most of the studied first‐line regimens; for most regimens, lower ECOG PS, younger age, female sex and adverse events were associated with longer survival.

**What this study adds:**

Real‐life effectiveness of certain treatment regimens may strongly differ in various populations/health care systems.

## Graphical abstract

Comparison between our TULUNG data and pooled survival data from clinical trials showed higher real‐life effectiveness for most of the studied first‐line regimens. We found that lower ECOG PS, younger age, female sex and adverse events were associated with longer survival. We demonstrated that real‐life effectiveness of certain treatments may strongly differ in various populations and health care systems.

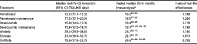

## Introduction

Treatment of advanced‐stage (IIIB/IV) non‐small cell lung cancer (NSCLC) has undergone notable evolution during the last 20 years. Antifolates, antiangiogenics, tyrosine kinase inhibitors (TKIs), anaplastic lymphoma kinase (ALK) inhibitors and immunotherapy have been introduced, with patients gaining a clear advance in quality of life, progression‐free survival (PFS) and overall survival (OS).^1^ In our previous study using data from the TULUNG Registry, we demonstrated that probability of two‐year survival of stage IIIB/IV patients with NSCLC doubled between 2011–12 and 2015–16.^2^ This observation may be explained by the introduction of several classes of new drugs in recent decades, together with the opportunity to introduce treatment multilinearity/sequencing in individual patients.^2^ Importantly, a positive response in patients after first‐line anticancer treatment appears to be the strongest predictor to continue therapy to subsequent lines, eventually resulting in longer overall patient survival.^3^, ^4^

Real‐life effectiveness of anticancer drugs (as well as of any other drugs) used in clinical practice should be routinely evaluated as it may significantly differ from drug efficacy observed in clinical trials. This is very important, especially in the advanced stages of lung cancer where any treatment failure may lead to rapid disease progression and early death. In a Dutch study by Cramer‐van der Welle *et al*., real‐life effectiveness of first‐line chemotherapeutic regimens and gefinib and erlotinib was assessed.^5^ The authors concluded that a significant gap between clinical efficacy and real‐life effectiveness existed with EE factors (efficacy/effectiveness) ranging from 0.52 to 0.87 for various treatment regimens.^5^ However, in this study, the reported N for gefitinib and erlotinib reached only 35 and 24 patients, respectively, leaving the validity of the results for TKIs disputable.^5^ In a different study, the authors estimated that the RCTs overestimate OS and PFS by ca 6% and 18%, respectively.^6^

Another issue is that real‐life patients frequently have various comorbidities, unstable brain metastases, polypharmacy or may be older, resulting in noninclusion in clinical trials. This may leave the majority of patients and their caregivers with uncertainity about the effectiveness of newly started treatments. For example, the study by Halpin *et al*. showed that approximately 27% of patients with chronic obstructive pulmonary disease (COPD) were found eligible to participate in a clinical trial on COPD treatment.^7^ For patients with NSCLC, Kawachi and colleagues found that around 60% of patients were ineligible for a clinical trial, mainly due to brian metastases, poor performance status or a respiratory disease.^8^

Exploiting the opportunities given by the existence of the TULUNG Registry, in this study we aimed to analyze real‐life effectiveness of TKIs, bevacizumab and pemetrexed as first‐line anticancer treatments in patients with stage IIIB/IV NSCLC. We hypothesized that real‐life effectiveness of first‐line treatments may be different (probably lower) than efficacy reported in previous clinical trials.

## Methods

### Study population

We analyzed prospective data from the TULUNG Registry (a joint registry of the Czech Society for Oncology, the Czech Pneumological Society, and Institute of Biostatistics and Analyses, Ltd.). The Czech TULUNG Registry covers data of patients with advanced‐stage (IIIB–IV) NSCLC receiving modern anticancer treatments (antifolates, biological agents and/or immunotherapy). Patient recruitment to this multicenter database has been provided in 11 tertiary‐ or university‐degree healthcare centers in Czechia since 1 July 2011 and is still ongoing. Written informed consent was signed by each patient participating in this study. The following data were collected: demography (sex, age, body mass index, height, weight, performance status), patient history (comorbidities, smoking history), lung cancer histology, cancer stage at time of diagnosis (according to the seventh TNM classification),^9^ results of molecular genetic testing (mainly mutation status of epidermal growth factor receptor gene), anticancer treatments use (including record on dosage, adverse events, reason of treatment discontinuation), thoracic surgery or radiotherapy, and survival data (OS, PFS). Patients’ participation in the study was completely voluntary. Of note, a patient's refusal to participate in the registry had no influence on anticancer treatment accessibility. All data were collected continuously, and actualized regularly on at least twice a year basis and anonymized.

In order to study the real‐life effectiveness of modern anticancer treatments (antifolates and biological agents), we analyzed data from the TULUNG Registry from 1 July 2011 (ie, the foundation of the registry) to 30 June 2018, to allow for meaningful survival analyses by 2 September 2019. Patients with NSCLC stage IIIB/IV (according to the seventh TNM)^9^ treated with first‐line gefitinib, erlotinib, afatinib, bevacizumab, bevacizumab maintenance, pemetrexed or pemetrexed maintenance were included in the analysis. Patients who underwent other treatments (osimertinib, ALK inhibitors, immunotherapy, etc) were excluded due to a low N that prevented a meaningful statistical analysis being performed.

### Literature search, calculation of clinical efficacy

A comprehensive and systematic literature search was performed in order to identify clinical trials suitable for calculation of comparator survival parameters (OS and PFS). We searched for phase III trials regarding clinical efficacy of each treatment regimen in the PubMed, Central and Medline databases. Comparator data search was limited up to 31 December 2019.

The search results were evaluated by two independent reviewers. First, the titles and article abstracts were screened, followed by full‐text reading of those selected. Only full‐text and main articles reporting definitive results on OS and/or PFS (supported by confidence intervals) of stage IIIB/IV patients with NSCLC were considered suitable for pooled survival calculation. Articles with results from only post hoc or subgroup analyses were excluded from our final analysis, as well as clinical trials phase <3, immature OS data, higher than first‐line treatment, end‐points of studies not focused on PFS or OS, and others. The final selection of articles eligible for a pooled OS and/or PFS calculation was discussed by two reviewers until a definitive consensus was reached.

Comparator pooled OS and PFS were calculated from OS and PFS data of eligible articles retrieved from literature search for each treatment regimen separately; further details are described in the section below.

### Statistical analysis

Each patient group with a specific treatment regimen was considered a separate subgroup for statistical analysis. Basic characteristics of each specific treatment cohort are described by descriptive statistics. Continuous parameters are described by means and 95% confidence intervals (CI) and by median with minimum and maximum values. Categorical parameters are presented as absolute and relative frequencies. Relative frequencies were calculated based on numbers of patients in the relevant subgroups.

Overall survival (OS) is defined as the time from initiation of first‐line anticancer treatment to the date of death (due to any cause). Progression‐free survival (PFS) is defined as the time from initiation of first‐line anticancer treatment to the date of first documented progression or death due to any cause. Treatment responses were assessed using the RECIST 1.1 criteria.^10^ OS and PFS were estimated using Kaplan‐Meier method and all the point estimates include a 95% CI.

The pooled estimates of median PFS and median OS from clinical trials were calculated as a weighted estimate of population medians, using this formula^11^:

$$m_{p}={\left(\sum_{i=1}^{k}\frac{w_{i}}{m_{i}}\right)}^{-1}$$

where *m*_*i*_ represents the median survival within each study population (*i* = 1,2,…*k*), *w*_*i*_ refers to the weight of each study and is equivalent to the sample size of each study divided by the total sample size.

A new term – “index of real‐life effectiveness” (IRE) was proposed for a calculated ratio of real‐life OS (or PFS) of an anticancer drug/drug regimen to its comparator pooled OS (or PFS) calculated from clinical trials.

Our results of the IRE analyses showed that a significant proportion of long‐term survivors existed in most of the studied treatment subgroups. We also decided to analyze the factors associated with exceptionally longer OS. For this purpose, data of patients surviving ≥110% of calculated reference OS (from clinical trials) were used, cutoffs for entering regression analyses were set as follows: afatinib ≥26.2 months (calculated OS from trials was 24.7 months), bevacizumab ≥14.7 months (calculated OS from trials was 13.4 months), bevacizumab maintenance ≥14.6 months (calculated OS from trials was 13.3 months), erlotinib ≥24.9 months (calculated OS from trials was 22.7 months), gefitinib ≥29.3 months (calculated OS from trials was 26.6 months), pemetrexed ≥11.2 months (calculated OS from trials was 10.2 months) and pemetrexed maintenance ≥14.9 months (calculated OS from trials was 13.5 months). Univariate and multivariate logistic regression models were constructed for all types of treatment regimens separately and for these variables: age, sex, ECOG PS, clinical stage at treatment initiation, adverse events and presence of comorbidities.

Statistical analyses were performed using IBM SPSS, Statistics (version 25.0) and R software (version 3.5.1).

### Ethics

The foundation of the TULUNG Registry was approved by the Institutional Ethics Committees of all participating centers (University Hospital Olomouc, University Hospital Pilsen, University Hospital Brno, University Hospital Hradec Kralove, University Hospital Motol [Prague], University Hospital Bulovka [Prague], Thomayer Hospital [Prague], Jihlava Hospital, Masaryk Memorial Cancer Institute, Masaryk Hospital [Usti nad Labem], Na Homolce Hospital [Prague] and VFN [Prague]). This study was approved by the Ethics Committee of the University Hospital Hradec Kralove on 11 May 2018; approval number: 201805 I134R.

## Results

Data of 2157 patients from the TULUNG Registry fulfilled the inclusion criteria and were eligible for further statistical analyses. Of these, 62 patients were treated with erlotinib, 147 with afatinib, 325 with gefitinib, 466 with bevacizumab (186 patients continued on bevacizumab maintenance), and 1157 with pemetrexed (255 patients continued on pemetrexed maintenance). Basic characteristics of each treatment subgroup are presented in Table 1.

**Table 1:** Basic characteristics of each treatment subgroup

	Pemetrexed	Pemetrexed ‐ maintenance	Bevacizumab	Bevacizumab ‐ maintenance	Afatinib	Erlotinib	Gefitinib
No of patients	1157	255	466	186	147	62	325
Age (years)							
Mean (95% CI)	63.1 (62.5–63.6)	62.1 (60.9–63.3)	61.0 (60.5–61.9)	62.3 (60.9–63.6)	62.8 (61.0–64.6)	64.7 (61.8–67.5)	67.4 (66.2–68.6)
Gender, N (%)							
Male	676 (58.4%)	140 (54.9%)	264 (56.7%)	103 (55.4%)	51 (34.7%)	17 (27.4%)	108 (33.2%)
BMI (kg/m^2^)							
Mean (95% CI)	26.3 (26.0–26.6)	26.8 (26.2–27.4)	26.4 (25.9–26.8)	26.0 (25.3–26.7)	25.9 (25.1–26.6)	26.1 (25.0–27.1)	26.2 (25.7–26.8)
Race, N (%)							
Caucasian	1132 (99.6%)	252 (98.8%)	462 (99.8%)	186 (100.0%)	145 (98.6%)	59 (95.2%)	315 (96.9%)
Mongolian	4 (0.4%)	0 (0.0%)	0 (0.0%)	0 (0.0%)	2 (1.4%)	3 (4.8%)	10 (3.1%)
Black	1 (0.1%)	3 (1.2%)	1 (0.2%)	0 (0.0%)	0 (0.0%)	0 (0.0%)	0 (0.0%)
Smoking status, N (%)							
Non‐smoker	252 (21.8%)	49 (19.2%)	108 (23.2%)	43 (23.1%)	74 (50.3%)	40 (64.5%)	183 (56.3%)
Smoker/ex‐smoker	905 (78.2%)	206 (80.8%)	358 (86.8%)	143 (76.9%)	73 (49.7%)	22 (45.5%)	142 (43.7%)
ECOG PS, N (%)							
0	224 (19.4%)	88 (34.5%)	146 (31.3%)	66 (35.5%)	44 (29.9%)	7 (11.3%)	60 (18.5%)
1	907 (78.4%)	167 (65.5%)	311 (66.7%)	117 (62.9%)	103 (71.1%)	46 (74.2%)	223 (68.6%)
≥2	26 (2.3%)	0 (0.0%)	9 (1.9%)	3 (1.6%)	0 (0.0%)	9 (14.5%)	42 (12.9%)
Stage, N (%)							
IIIB	145 (12.5%)	35 (13.7%)	26 (5.6%)	15 (8.1%)	12 (8.2%)	8 (12.9%)	31 (9.5%)
IV	1012 (87.5%)	220 (86.3%)	440 (94.4%)	171 (91.9%)	135 (91.8%)	54 (87.1%)	294 (90.5%)
Comorbidity, N (%)							
Yes	51 (4.4%)	20 (7.8%)	26 (5.6%)	12 (6.5%)	9 (6.1%)	0 (0.0%)	21 (6.5%)
Histology, N (%)							
Adenocarcinoma	1096 (94.7%)	251 (98.4%)	439 (94.2%)	181 (97.3%)	145 (98.6%)	59 (95.2%)	307 (94.5%)
Squamous cell carcinoma	6 (0.5%)	0 (0.0%)	0 (0.0%)	0 (0.0%)	0 (0.0%)	1 (1.6%)	2 (0.6%)
Other type	55 (4.8%)	4 (1.6%)	27 (5.8%)	5 (2.7%)	2 (1.4%)	2 (3.2%)	16 (4.9%)
EGFR, N (%)							
Positive	36 (7.5%)	12 (5.0%)	10 (2.8%)	3 (1.9%)	147 (100.0%)	62 (100.0%)	325 (100.0%)
Status of therapy, N (%)							
Ongoing	24 (2.1%)	7 (2.8%)	3 (0.6%)	4 (2.2%)	32 (21.8%)	7 (11.3%)	26 (8.0%)
Terminated	1133 (97.9%)	248 (97.3%)	463 (99.4%)	182 (97.8%)	115 (78.2%)	55 (88.7%)	299 (92.0%)
Adverse event, N (%)							
Yes	270 (23.3%)	14 (5.5%)	44 (9.4%)	7 (3.8%)	63 (42.9%)	27 (43.6%)	90 (27.7%)

95% CI, 95% confidence interval; ECOG PS, Eastern Cooperative Oncology Group Performance Status; EGFR, epidermal growth factor receptor; kg, kilogram; m^2^, square meter; N, number of patients.

Survival analysis from the TULUNG cohort showed the following results for the studied treatment subgroups (displayed as median): erlotinib – PFS 12.2 months and OS 23 months, afatinib – PFS 13.1 months and OS 29.3 months, gefitinib – PFS 10.4 months and OS 19.6 months, pemetrexed – PFS 4.9 months and OS 12.2 months, pemetrexed maintenance – PFS 5.5 months and OS 17.5 months, bevacizumab – PFS 5.6 months and OS 15.8 months, bevacizumab maintenance – PFS 4.5 months and OS 15.8 months. Complete survival data including 95% confidence intervals are presented in Tables 2 and 3.

**Table 2:** Overall survival of each treatment subgroup from the TULUNG Registry

Treatment	Median real‐life OS in months (95% CI) (TULUNG data)	Pooled median OS in months (metaanalysis)*	Index of real‐life effectiveness
Pemetrexed	12.2 (11.1–13.5)	10.2^21^, ^22^, ^23^, ^24^, ^25^	1.188
Pemetrexed maintenance	17.5 (15.1–20.3)	13.5^17^, ^18^, ^19^, ^20^	1.294
Bevacizumab	15.8 (14.0–17.3)	13.4^26^, ^27^, ^28^	1.178
Bevacizumab maintenance	15.8 (13.9–18.7)	13.3^19^, ^20^, ^29^, ^30^	1.189
Afatinib	29.3 (20.9–38.6)	24.7^40^, ^41^	1.184
Erlotinib	23.0 (18.9–32.7)	22.7^42^, ^43^	1.013
Gefitinib	19.6 (17.6–23.2)	26.6^34^, ^35^, ^36^, ^44^, ^45^, ^46^, ^47^, ^48^, ^49^	0.736

95% CI, 95% confidence interval; OS, overall survival.

* Note: for reference pooled OS, 95% CIs were not calculable since some of the reference studies did not include data on 95% CIs.

**Table 3:** Progression‐free survival of each treatment subgroup from the TULUNG Registry

Treatment	Median real‐life PFS in months (95% CI) (TULUNG data)	Pooled median PFS in months (metaanalysis)*	Index of real‐life effectiveness
Pemetrexed	4.9 (4.5–5.3)	5.0^22^, ^23^, ^24^, ^25^	0.972
Pemetrexed maintenance	5.5 (4.7–6.2)	4.4^17^, ^18^, ^19^, ^20^	1.252
Bevacizumab	5.6 (5.1–6.3)	8.3^26^, ^27^, ^28^	0.676
Bevacizumab maintenance	4.5 (3.9–5.1)	4.6^19^, ^20^, ^29^, ^30^	0.99
Afatinib	13.1 (11.4–18.2)	11.0^40^, ^41^	1.189
Erlotinib	12.2 (9.1–17.3)	10.8^42^, ^50^, ^51^, ^52^, ^53^	1.123
Gefitinib	10.4 (8.9–11.3)	7.9^36^, ^40^, ^44^, ^45^, ^46^, ^47^, ^48^, ^49^, ^53^, ^54^, ^55^, ^56^	1.314

95% CI, 95% confidence interval; PFS, progression‐free survival.

* Note: for reference pooled PFS, 95% CIs were not calculable since some of the reference studies did not include data on 95% CIs.

Calculated (pooled) OS and PFS from clinical trials for each treatment regimen are presented in Tables 2 and 3. Calculated IREs for particular treatment regimens were: erlotinib – 1.123 for PFS and 1.013 for OS, afatinib – 1.189 for PFS and 1.184 for OS, gefitinib – 1.314 for PFS and 0.736 for OS, pemetrexed – 0.972 for PFS and 1.188 for OS, pemetrexed maintenance – 1.252 for PFS and 1.294 for OS, bevacizumab – 0.676 for PFS and 1.178 for OS, and bevacizumab maintenance – 0.99 for PFS and 1.189 for OS (Tables 2 and 3).

Multivariate logistic regression models showed these factors independently and significantly associated with longer OS (110% of pooled reference OS from clinical trials for each regimen separately): lower PS for afatinib; lower PS, absence of adverse events and female sex for bevacizumab; lower PS and female sex for pemetrexed. Complete results of univariate and multivariate regression models are presented in Table 4.

**Table 4:** Results of univariate and multivariate analyses for patients surviving ≥110% of calculated pooled reference OS (= with IRE ≥1.1)

			Univariate analysis		Multivariate analysis	
Treatment	Variable	N (%) for categorical variables Median (Min‐Max) for continuous variables	OR (95% CI)	*P*‐value	OR (95% CI)	*P*‐value
Pemetrexed	**PS**					
	0	174 (19.0%)	Reference category	—	Reference category	—
	1	718 (78.5%)	0.38 (0.27–0.53)	**<0.001**	0.38 (0.26–0.53)	**<0.001**
	2	23 (2.5%)	0.25 (0.09–0.63)	**0.004**	0.25 (0.09–0.63)	**0.004**
	**Sex**					
	Men	529 (57.8%)	Reference category	—	Reference category	—
	Women	386 (42.2%)	1.58 (1.22–2.07)	**0.001**	1.62 (1.23–2.12)	**0.001**
Pemetrexed maintenance	—	—	—	—	—	—
Bevacizumab	**PS**					
	0	123 (34.6%)	Reference category	—	Reference category	—
	1	224 (62.9%)	0.34 (0.22–0.54)	**<0.001**	0.33 (0.20–0.52)	**<0.001**
	2	9 (2.5%)	0.18 (0.03–0.79)	**0.039**	0.24 (0.03–1.08)	0.088
	**Sex**					
	Men	203 (57.0%)	Reference category	—	Reference category	—
	Women	153 (43.0%)	1.96 (1.28–3.01)	**0.002**	1.74 (1.11–2.75)	**0.017**
	**Adverse events**					
	No	318 (89.3%)	Reference category	—	Reference category	—
	Yes	38 (10.7%)	0.49 (0.23–1.00)	0.059	0.39 (0.17–0.84)	**0.020**
Bevacizumab maintenance	—	—	—	—	—	—
Afatinib	**PS**					
	0	30 (34.9%)	Reference category	—	Reference category	—
	1	56 (65.1%)	0.26 (0.10–0.67)	**0.006**	0.27 (0.09–0.73)	**0.011**
Erlotinib	**Age**	67.5 (39.7–84.6)	1.07 (1.01–1.14)	**0.046**	1.06 (0.99–1.15)	0.092
	**Adverse events**					
	No	23 (50.0%)	Reference category	—	Reference category	—
	Yes	23 (50.0%)	5.18 (1.42–22.36)	**0.017**	3.31 (0.73–16.84)	0.127
Gefitinib	—	—	—	—	—	—

Regression models were constructed for each regimen separately. Variables entered into the analyses were the same for all regimens, including: age, sex, ECOG PS, clinical stage at treatment initiation, adverse events and presence of comorbidities. Table 4 contains only results reaching statistical significance. 95% CI, 95% confidence interval; IRE, Index of Real‐life Effectiveness; Max, maximum; Min, minimum; N, number of patients; OR, odds ratio; PS, Eastern Cooperative Oncology Group Performance Status.

## Discussion

We present the results of one of the largest studies on real‐life effectiveness of modern‐era first‐line anticancer treatments in patients with stage IIIB/IV NSCLC. The study cohort is robust (2157 patients) and reflects the therapeutic impact of various treatment regimens in an era that preceded the routine clinical use of immunotherapy, ALK inhibitors and combined multiclass drug regimens. Our data analysis ended with patients who were being treated with first‐line treatment initiated up to 30 June 2018. Since then, several newer drugs including ALK inhibitors: crizotinib, alectinib; TKIs: osimertinib; immunotherapy: nivolumab, pembrolizumab, and durvalumab maintenance have been approved for clinical use in Czechia. With regard to this study, the number of patients in these new groups were small and treatment duration was too short for a meaningful survival analysis.

For comparison of real‐life effectiveness of an anticancer drug (or a drug regimen) with its efficacy known from clinical trials, we proposed the term “index of real‐life effectiveness (IRE)”, a ratio of real‐life OS (or PFS) and a pooled OS (or PFS) calculated from clinical trials. IRE represents a simple mode to express how treatment efficacy from clinical trials translates into daily clinical practice. Obviously, this parameter may reach different values depending on the study population and period. In turn, the values of IRE (particularly those lower than 1.0) may be a very important feedback for clinicians taking care of lung cancer patients.

A similar attempt to express real‐life effectiveness was done by Dutch authors from the Santeon group.^5^ In their study, a pooled efficacy/effectiveness factor of 0.77 for all the studied treatments on a cohort of 1122 metastatic‐stage NSCLC patients was observed.^5^ Cramer‐van der Welle and her colleagues assumed that an approximate 25% lower effectiveness of anticancer treatments (compared to trials) should be expected during clinical decision‐making. The authors of that study concluded that the reduced effectiveness is a constant pattern irrespective of type of treatment regimen. These observations are, however, in contrast with our findings where real‐life effectiveness for OS of most of the studied regimens turned out to be even higher (ie, with IRE >1) than comparator efficacy from trials. This is clear evidence that parameters such as the IRE (or EE factor) perform unequally in different populations and the results and conclusions of the Dutch study cannot be extrapolated to all countries and their healthcare systems. Moreover, IRE or EE factor is not rigid and may be subject to changes over time. Clinical decision‐making with the aid of similar parameters should be performed with caution and respect to local healthcare systems.

We propose several explanations for the discrepancy between our findings and the observations made by the Dutch authors in the Cramer‐van der Welle study. First, in the Dutch study, some of the specific treatment subgroups consisted of only a few patients (ie, *n* = 35 for gefitinib and *n* = 24 for the erlotinib subgroups). Second, differences may exist between the Czech and the Dutch health care systems regarding care for lung cancer patients. Third, there is a liberal attitude towards euthanasia in the Netherlands. Deaths from lung cancer annually account for approximately 10–11 000 of all deaths in the Netherlands.^12^ Of these, ~10% have been reported to be attributable to euthanasia.^13^ Replacement of further treatment lines or best supportive care after disease progression by the act of euthanasia may result in shorter survival. Importantly, in their study, Pardon *et al*. found that of the 13 lung cancer patients that first requested euthanasia but finally did not end up with it, eight patients subsequently had “alleviated symptoms”.^14^ We speculate that the euthanasia issue might also bias the survival results in the Cramer‐van der Welle study (median OS for the complete cohort was only 8.02 months).^5^ This issue has not been specifically addressed by the authors of that study.^5^ In contrast, our results showed a pooled median OS of 15.3 months.

Another potential contributor to better survival in our study was the increased chance of treatment multilinearity/sequencing. In our previous study from the TULUNG Registry, treatment multilinearity was more frequent in recent years (1.54 line per patient in 2015–16 compared to 1.48 line in 2011–12) and associated with longer OS in patients with lung adenocarcinoma.^2^ The effect was more prominent with increasing number of treatment lines.^2^ Better survival outcomes in more recent patients (compared to previous years) were also observed in a Swedish study.^15^ Importantly, three studies showed that a success within first‐line therapy also positively affected outcomes in subsequent treatment lines.^3^, ^4^, ^16^

Both bevacizumab‐ and pemetrexed‐based regimens had good clinical effect on OS (IREs were ≥1.17) in our patients not harboring any clinically relevant mutation. Both these regimens present effective tools for improving survival measures.^17^, ^18^, ^19^, ^20^, ^21^, ^22^, ^23^, ^24^, ^25^, ^26^, ^27^, ^28^, ^29^, ^30^ In an Italian real‐life study performed on only 62 NSCLC patients, median OS for first‐line bevacizumab maintenance treatment was 10.5 months.^31^ Median PFS was 7.4 months for bevacizumab‐based therapy while 6.4 months for pemetrexed in a Taiwanese study.^32^ Another interesting finding was that patients completing maintenance‐type regimens had higher IRE for OS than those receiving conventional regimens of treatment. These data may simply reflect the proportion of well‐responders in the cohort, in our case it was around 20%–24%.

In our EGFR‐positive patients, afatinib was the most effective drug regarding effect on PFS and OS. This is partly due to treatment restriction in the first years of its use (afatinib was approved only for patients with ECOG PS 0‐1) and younger age of patients. Our data showed that gefinitib had lowest IRE for OS (0.736) of the three studied TKIs, while IRE for PFS was 1.314. The main reason for this discrepancy might be that gefitinib has a more favorable safety profile and is given to fragile, older patients with NSCLC.^33^ In our cohort, patients treated by gefitinib were oldest (mean age: 67.4 years) of all TKIs groups but had lowest overall adverse event rate (27.7%) compared to erlotinib and afatinib (43.6% vs. 42.9%). However, the frailty of these patients may result in lower rates of subsequent treatment lines and shorter OS. Our comparator OS was also calculated on more trials´ data; recently, longer OS for gefitinib was reported than previously.^34^, ^35^, ^36^ The real‐life OS for gefitinib was about similar in our (19.6 months) and in the Dutch study (21.19 months).^5^ In a real‐life Taiwanese study, the observed PFS and OS were 11.9 and 26.9 months, respectively.^37^ Comparable to our results, in a Californian study, TKIs had the most prominent effect on OS among all the studied treatments in stage IV nonsquamous NSCLC patients.^38^

We observed certain discrepancies between IREs for OS and PFS in various studied regimens (IRE for PFS was <1 but IRE for OS was >1 for pemetrexed, bevacizumab and bevacizumab maintenance). We speculate that the PFSs might be subject to bias by different intervals between CT scans performed during real‐life treatment and clinical trials. On the other hand, OS data are more precise and as such, should be considered an objective measure of treatment success in our cohort. In Czechia, approximately 80% of center care for lung cancer patients is concentrated in the hands of pneumologists/pneumooncologists. We speculate that a pulmonologist's concentrated and timely management of not only lung cancer, but also pulmonary comorbidities (COPD, pleural effusions, hemoptysis, endobronchial procedures, pulmonary embolism, pulmonary adverse events of treatments etc) and other supportive care may preserve good ECOG PS enabling the patient to enter subsequent lines of treatment, which will eventually result in longer OS. Although treatment selection and initiation should be guided by scientific evidence, in the real‐life setting, selection of patients for certain treatment may slightly differ. Therefore, we cannot completely rule out selection bias in our OS results.

OS data were surprisingly good in our cohort. With special emphasis on long‐term survivors, the results of the regression models showed constant patterns across all treatment subgroups: lower PS, lower disease stage, female sex, younger age and less comorbidity tended to be associated with longer OS (≥110% of calculated pooled OS from trials). However, most of these findings did not reach statistical significance due to low N in the specific subgroup. Of note, the presence of adverse events in bevacizumab‐treated patients tended to result in a worse prognosis (OR 0.39; 95% CI: 0.17–0.84; *P* = 0.02), while for TKIs‐based regimens, the presence of adverse events was associated with higher probability of longer OS. This observation was more pronounced in the erlotinib subgroup (OR 3.31; 95% CI: 0.73–16.84; *P* = 0.127). In accordance with our results, younger age, less advanced disease stage and less comorbidity were associated with a more favorable outcome in TKI‐treated NSCLC patients in a Swedish observational study.^15^

### Strengths and limitations

The main strength of our study is that the data are very robust (*n* = 2157) and fully representative of the real‐life situation in Czechia. The data were collected in nine university‐type centers providing specialized pneumooncology care. Modern‐era anticancer treatments for NSCLC patients (biologicals, antiangiogenics, antifolates, immunotherapy) may only be given at these specialized centers.

There were several limitations in our study. First, for erlotinib, the number of patients in this group was rather lower (*n* = 62) and validity of survival analysis for this subgroup may be limited. Second, the TULUNG is a registry of patients treated by modern‐era drugs only (antifolates, TKIs, antiangiogenics, ALK inhibitors, immunotherapy) while data of patients treated by a first‐line conventional chemotherapy regimen are not included. This means that the majority (>90%) of patients in the TULUNG Registry are adenocarcinoma‐histology patients. Third, the data are derived from a national multicenter registry. Therefore, we are unable to confirm that the TULUNG Registry contains complete data of all NSCLC patients from Czechia since data of a minority of patients may be missing (eg, patient's disagreement with inclusion in the Registry, data entry bias etc). Fourth, some patients were alive at the time of data analysis. However, the proportion of alive patients was only approxmately 4% of the study cohort and the results are unlikely to be biased to a large extent. Fifth, for pemetrexed‐based regimens, we did not differentiate between carboplatin and ciplatin since the survival effect of these drugs is almost identical.^5^, ^39^, ^40^ Only a small percentage of patients received carboplatin. Therefore, the risk of bias on OS is extremely low. Also, our study did not primarily focus on side effects. Sixth, at the time of writing this manuscript, ALK inhibitors: crizotinib, alectinib; TKIs: osimertinib; immunotherapy: nivolumab, pembrolizumab, durvalumab maintenance were already approved by Czech State Institute for Drug Control as first‐line drugs for treatment of NSCLC in Czechia. However, due to their late approvals as first‐line drugs/regimens, the TULUNG Registry did not include sufficient data of patients with mature OS (eg, for pembrolizumab, two‐year survival data were collected only from three patients by the end of 2019). Therefore, we were unable to provide a meaningful statistical analysis regarding real‐life efficacy of these drugs. It will be interesting to perform a similar analysis for the aforementioned drugs/regimens in the coming 2–3 years, although, with regard to immunotherapy, the currently available real‐life evidence seems to be sufficient at this time. Finally, systematic assessment of quality of life measures is not part of the TULUNG Registry. However, ECOG PS data of our cohort are fully available.

In conclusion, we found higher real‐life effectiveness of most first‐line anticancer treatments used in stage IIIB/IV NSCLC therapy, compared to pooled efficacy calculated from clinical trials. As such, our data show conflicting results with those from the Dutch Cramer‐van der Welle study where a significantly lower efficacy/effectiveness factor (0.77) for all studied treatments was observed. Whilst we do not contest the results of the Dutch study, in this study we clearly demonstrate that parameters such as the IRE (or EE factor) may perform unequally in different populations and the results and conclusions of a single study cannot be extrapolated to all countries and their respective healthcare systems.

In *EGFR* wild‐type patients, bevacizumab‐based regimens had the most prominent effect on OS of the studied treatments. In relative values (IRE), however, pemetrexed‐based regimens performed even better (IRE 1.188 and 1.294). Of the TKIs, afatinib was by far the most effective drug for OS in *EGFR*‐positive NSCLC patients (OS 29.3 months, IRE 1.184).

## Disclosure

The authors of this manuscript report no conflicts of interest with regard to this study.

## Supporting information


**Appendix S1:** Supplementary Information.
