# Supplementary material for: Real‐life effectiveness of first‐line anticancer treatments in stage IIIB/IV NSCLC patients: Data from the Czech TULUNG Registry
Source: Thorac Cancer. 2020 Oct 5;11(11):3346–56. doi: 10.1111/1759-7714.13679 (PMC7606010; doi:10.1111/1759-7714.13679)
Supplement: Supplementary file 1 — Appendix S1: Supplementary Information. [file TCA-11-3346-s001.pdf]

Supplementary Figure 1.
Literature search and final selection of studies for regimen: **Platinum derivative + Pemetrexed**

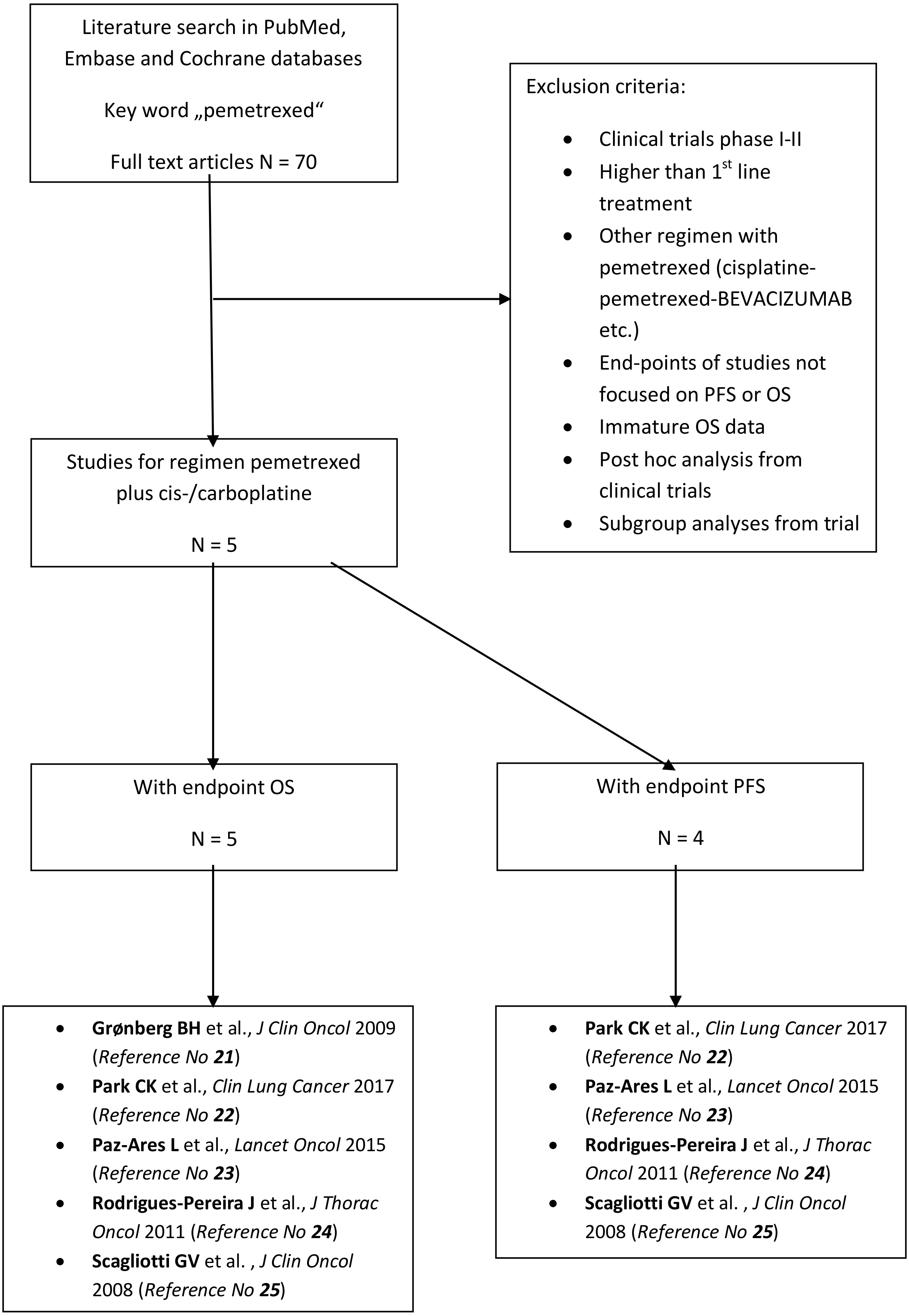

Supplementary Figure 2.

Literature search and final selection of studies for regimen: **Platinum derivative + Pemetrexed, followed by Pemetrexed maintenance**

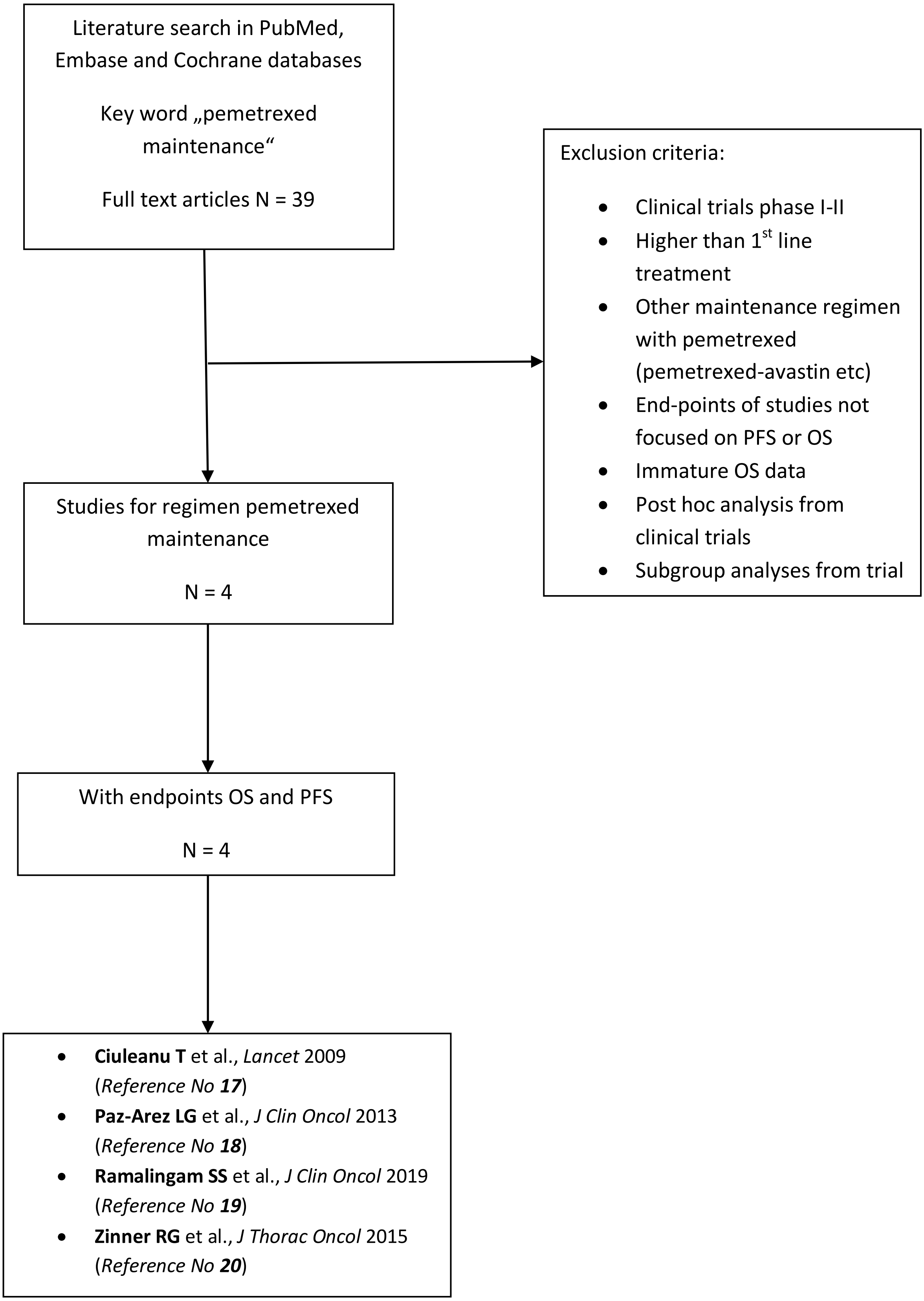

Supplementary Figure 3.
Literature search and final selection of studies for regimen: **Bevacizumab**

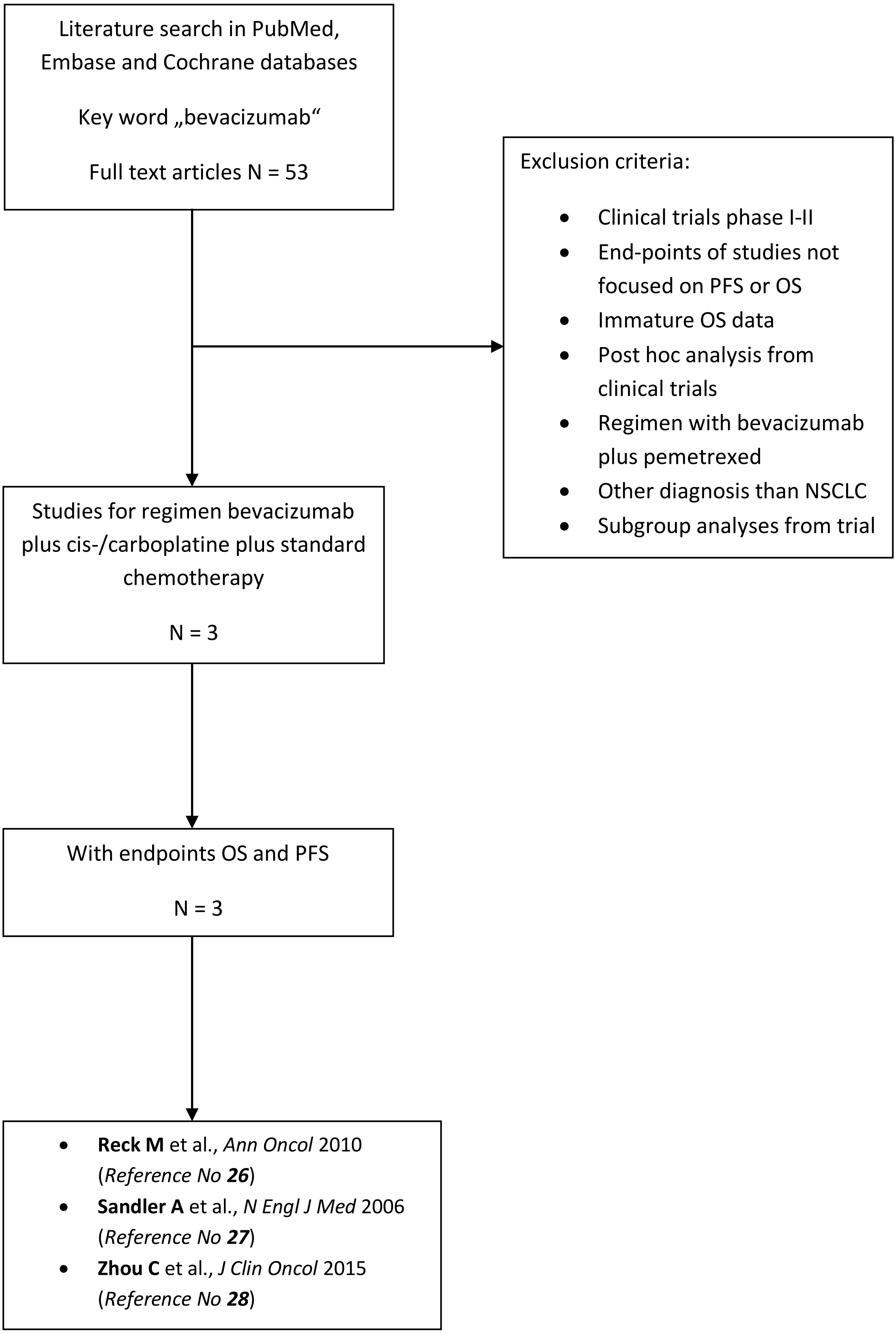

Supplementary Figure 4.

Literature search and final selection of studies for regimen: **Bevacizumab maintenance**

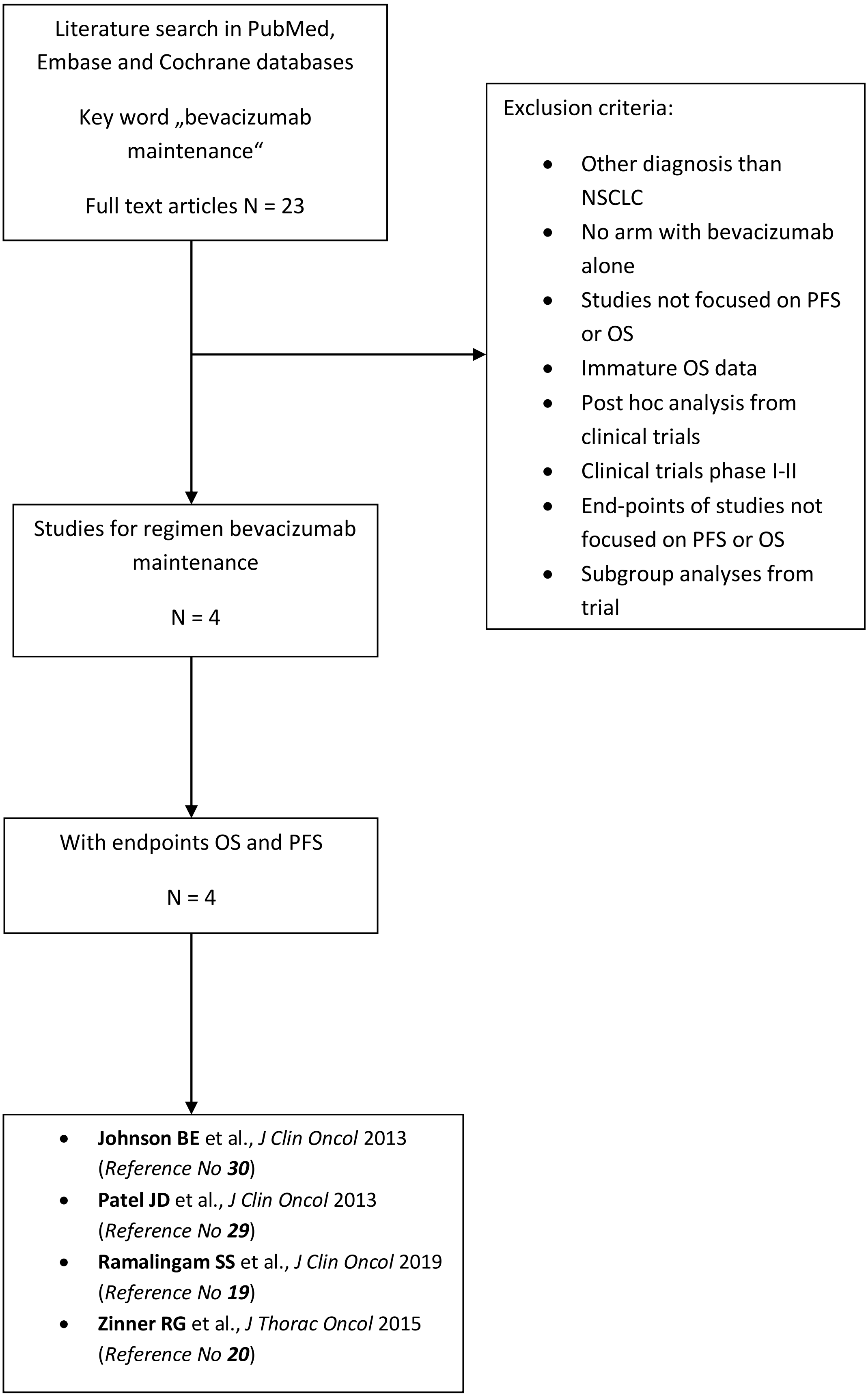

Supplementary Figure 5.
Literature search and final selection of studies for regimen: **Erlotinib**

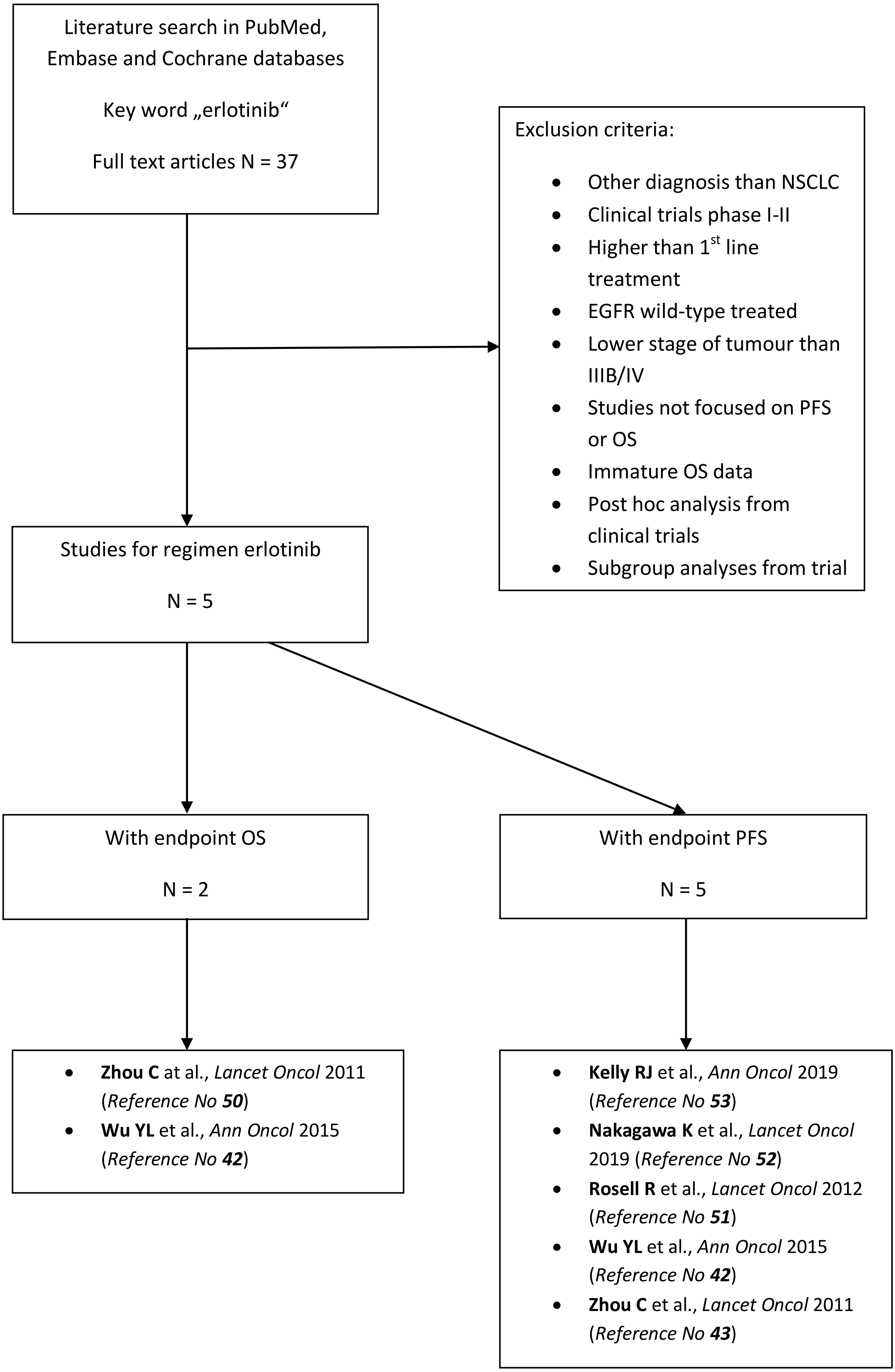

Supplementary Figure 6.
Literature search and final selection of studies for regimen: **Afatinib**

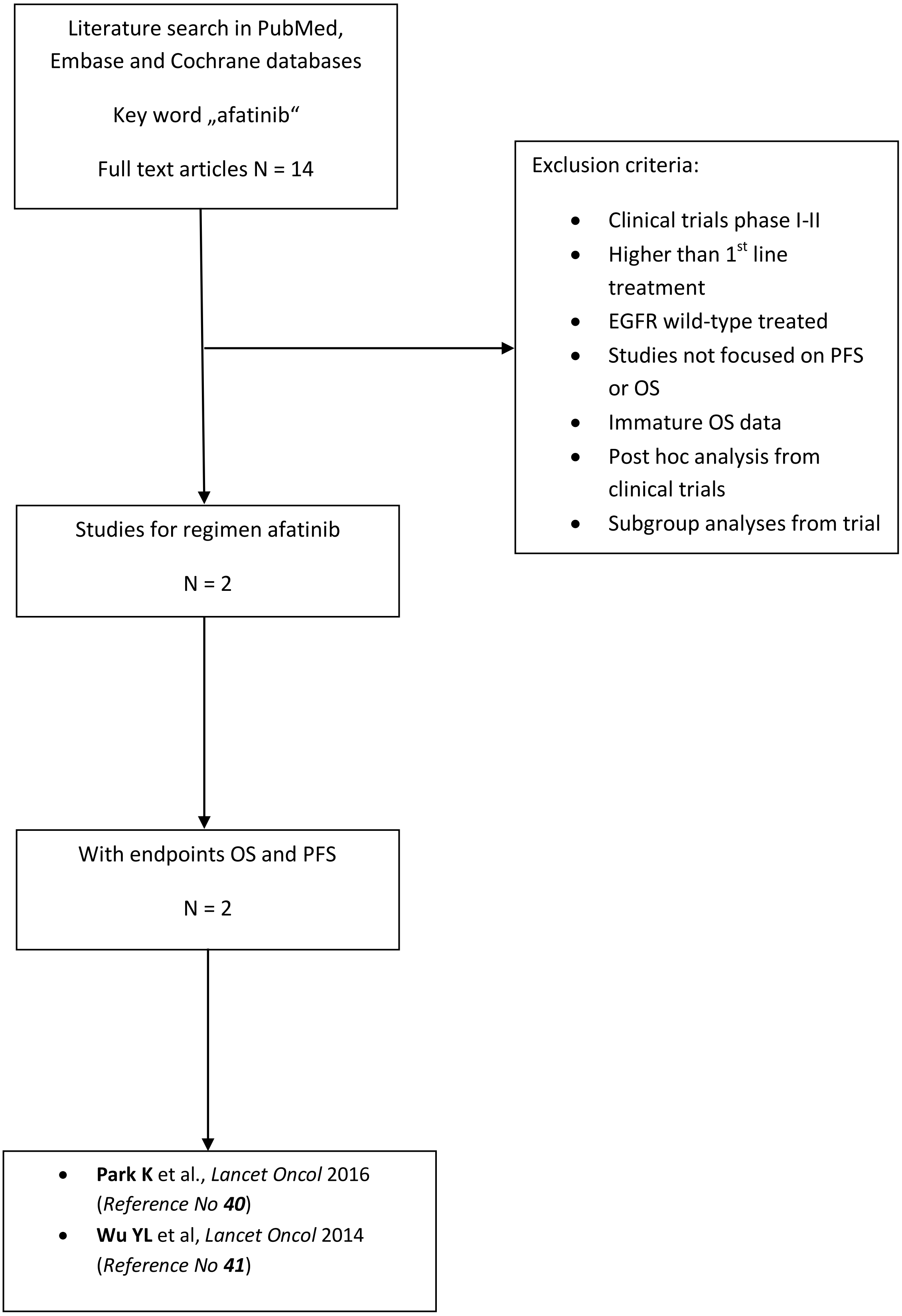

Supplementary Figure 7.
Literature search and final selection of studies for regimen: **Gefitinib**

Literature search in PubMed, Embase and Cochrane databases

Key word „gefitinib“

Full text articles N = 33

Exclusion criteria:

- Clinical trials phase I a II
- EGFR wild-type treated
- Lower stage than IIIB/IV
- Studies not focused on PFS or OS
- Immature OS data
- Post hoc analysis from clinical trials
- Subgroup analyses from trial

Studies for regimen gefitinib

N = 13

With endpoint OS

N = 9

- **An C** et al., *Pathol Oncol Res* 2016 (*Reference No **44***)
- **Han B** et al., *Int J Cancer* 2017 (*Reference No **45***)
- **Han JY** at al., *J Clin Oncol* 2012 (*Reference No **46***)
- **Hosomi Yl** et al., *J Clin Oncol* 2020 (*Reference No **36***)
- **Inoue A** et al., *Ann Oncol* 2013 (*Reference No **47***)
- **Maemondo M** et al., *N Engl J Med* 2010 (*Reference No **48***)
- **Mok TS** et al., *N Engl J Med* 2009 (*Reference No **55***)
- **Patil V** et al., *ESMO Open* 2017 (*Reference No **49***)
- **Yoshioka H** et al., *Ann Oncol* 2019 (*Reference No **34***)

With endpoint PFS

N = 12

- **An C** et al.,*Pathol Oncol Res* 2016 (*Reference No **44***)
- **Han B** et al., *Int J Cancer* 2017 (*Reference No **45***)
- **Han JY** at al., *J Clin Oncol* 2012 (*Reference No **46***)
- **Hosomi Yl** et al., *J Clin Oncol* 2020 (*Reference No **36***)
- **Inoue A** et al., *Ann Oncol* 2013 (*Reference No **47***)
- **Kelly RJ** at al. *Ann Oncol* 2019 (*Reference No **53***)
- **Maemondo M** et al., *N Engl J Med* 2010 (*Reference No **48***)
- **Mitsudomi T** et al., *Lancet Oncol* 2010 (*Reference No **54***)
- **Mok TS** et al., *N Engl J Med* 2009 (*Reference No **55***)
- **Park K** et al., *Lancet Oncol* 2016 (*Reference No **40***)
- **Patil V** et al., *ESMO Open* 2017 (*Reference No **49***)
- **Wu YL** et al., *Lancet Oncol* 2017 (*Reference No **56***)
